# Space-to-ground infrared camouflage with radiative heat dissipation

**DOI:** 10.1038/s41377-025-01824-y

**Published:** 2025-03-26

**Authors:** Bing Qin, Huanzheng Zhu, Rongxuan Zhu, Meng Zhao, Min Qiu, Qiang Li

**Affiliations:** 1 State Key Laboratory of Extreme Photonics and Instrumentation, College of Optical Science and Engineering, Zhejiang University, Hangzhou, 310027 China; 2 Key Laboratory of 3D Micro/Nano Fabrication and Characterization of Zhejiang Province, School of Engineering, Westlake University, Hangzhou, 310024 China

**Keywords:** Metamaterials, Mid-infrared photonics

## Abstract

With the development of space exploration and exploitation, it is imperative to address the potential threats posed to space objects, particularly ground-based infrared observation. However, in the extreme space environment, achieving infrared camouflage across different bands with simultaneous thermal management is challenging and has so far slipped out of concern. Here, we propose the space-to-ground infrared camouflage strategy, compatible with radiative heat dissipation. Camouflage in the H, K, mid-wave-infrared (MWIR), and long-wave-infrared (LWIR) bands is achieved through a multilayer structure, with radiative heat dissipation in the very-long-wave-infrared (VLWIR) band. High absorptivity (0.839/0.633) in the H/K bands minimizes the reflected signal of solar radiation and low emissivity (0.132/0.142) in the MWIR/LWIR bands suppresses the thermal radiation signal. Additionally, high emissivity (0.798) in the VLWIR band ensures efficient thermal management, resulting in a temperature decrement of 39.8 °C to the metal reference in the simulated space environment (with 1200 W m^−^^2^ thermal input). This work inspires sophisticated spectral manipulation in extreme environments and guides the development of camouflage and radiative heat dissipation techniques for space objects.

## Abstract (web summary)

Space-to-ground infrared camouflage with radiative heat dissipation is achieved via novel multilayer structure.

## Graphical abstract

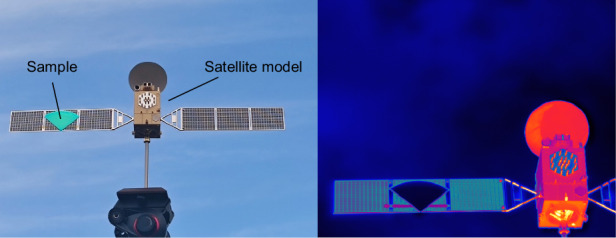

## Introduction

**Fig. 1:**
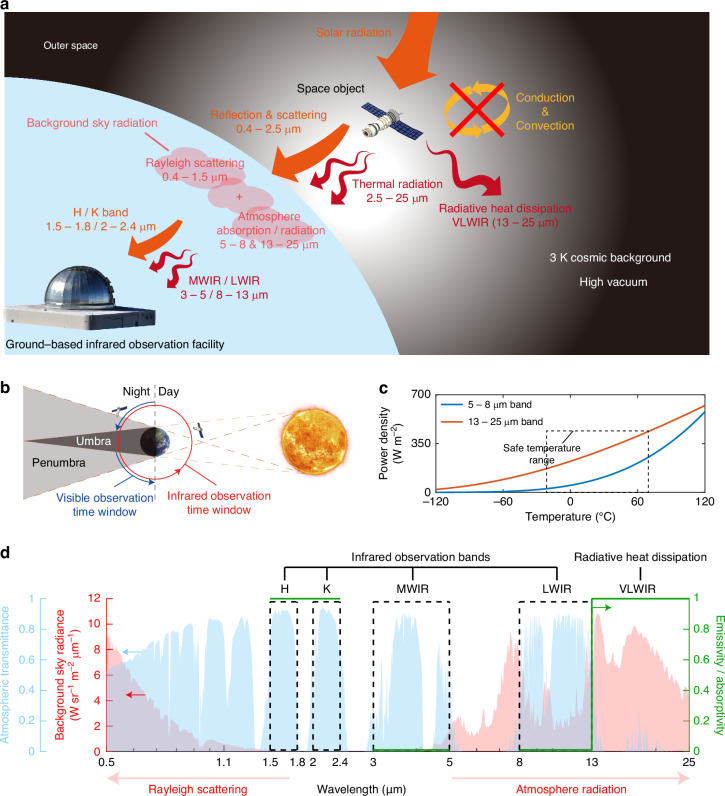
**Principle for space-to-ground infrared camouflage with radiative heat dissipation**. **a** Two kinds of infrared signals of space objects, reflection & scattering of solar radiance and thermal radiation, are detected by ground-based infrared observation facilities, after traveling through the atmosphere. Conduction & Convection are inhibited in outer space, leaving thermal radiation as the sole way for heat dissipation. **b** Comparison of ground-based infrared and visible observation facilities. **c** Comparison of the radiative heat dissipation within the 5–8 μm and 13–25 μm bands. **d** The most threatening detection bands of ground-based infrared observation facilities, considering the background sky radiation (red area) and atmosphere transmittance (blue area), and the ideal emissivity/absorptivity spectrum (green line) for space-to-ground infrared camouflage with radiative heat dissipation

Exploring space has been a perpetual endeavor for humanity. Thanks to the advancements in aerospace technology, the prospect of exploring and exploiting space is no longer an elusive dream. To date, over 12,000 spacecrafts have been launched into space, with the number increasing at an exponential rate in recent years. These diverse spacecrafts serve various aspects of human life and provide numerous conveniences. However, space objects are subjected to the extreme conditions of space and face threats from terrestrial equipment, notably detection by ground-based observation facilities (Fig. 1a). Among these facilities, the applicability of microwave facilities is confined to the surveillance of objects within low Earth orbit, with a limited resolution; visible facilities face constraints during daytime and cannot monitor space objects as they traverse into the Earth’s umbra, where no visible signals are reflected by objects^1^ (Fig. 1b). Conversely, infrared facilities effectively compensate for the limitations of visible observation facilities in terms of observation time and microwave observation facilities in terms of observation distance and resolution. Hence, the capability of infrared facilities for near-continuous, high-resolution detection severely threatens objects in various orbits. Based on their operational principle, infrared observation facilities can be classified into two categories: those that operate within the 1.1–2.5 μm band to capture solar radiation reflected by the space object, and those that function within the 2.5–25 μm band to detect the thermal radiation emitted by the space object (Fig. 1a). Consequently, there is a pressing need to develop space-to-ground infrared camouflage technologies to mitigate these threats and ensure the safe operation of space objects.

Infrared camouflage for aerial and terrestrial objects has witnessed substantial advancements, encompassing the utilization of metallic/dielectric structures^2–24^, and thermochromic^25–31^/electrochromic^32–37^ materials to effectively manipulate the infrared spectrum. In addition, the application of spectral-selective emitters has been instrumental in facilitating radiative heat dissipation within non-atmospheric windows (e.g., 2.5–3 μm^38,39^, 5–8 μm^38–50^, and 13–16 μm^44,45^), through photonic crystals^38,41,43–45,51^, metasurfaces^46–49^, and Fabry-Perot cavities^39^. Despite the advancements in infrared camouflage technologies, their effectiveness remains limited in the unique and extreme environment of space. Firstly, infrared camouflage in the solar radiation bands, especially the H (1.5–1.8 μm) and K (2–2.4 μm) bands, is rarely considered. A high signal-to-noise ratio can be achieved by ground-based observation facilities in the H/K bands, posing great threats to space objects. Secondly, the efficacy of current radiative heat dissipation bands is insufficient for space objects to maintain a safe temperature range (typically −20 to 70 °C)^52^ (Fig. 1c), calling for more appropriate heat dissipation bands. In space environment, both conduction and convection are inhibited, leaving thermal radiation as the sole way for heat dissipation^53–62^ (Fig. 1a). Finally, the extreme application scenario in space necessitates the development of camouflage materials with reduced weight and enhanced robustness. Therefore, achieving space-to-ground infrared camouflage across different bands with simultaneously radiative heat dissipation remains a challenge.

In this work, we propose a strategy that realizes space-to-ground camouflage in threatening infrared bands (H, K, mid-wave-infrared (MWIR, 3–5 μm), and long-wave-infrared (LWIR, 8–13 μm)), with concurrently radiative heat dissipation in the very-long-wave-infrared band (VLWIR, 13–25 μm). Firstly, high absorptivity (0.839/0.633) is achieved in the H/K bands to minimize the reflected signal of solar radiation. Secondly, reduced emissivity (0.132/0.142) in the MWIR/LWIR bands is realized to suppress thermal radiation signals from space objects. The camouflage performance in these bands is further demonstrated through simulated ground-based detection scenarios using infrared imagers. Thirdly, high emissivity (0.798) is achieved in the VLWIR band for radiative heat dissipation, contributing to a temperature decrement of 39.8 °C to the metal reference in the simulated space environment (with an input thermal power of 1200 W m^−^^2^). This work inspires sophisticated spectral manipulation in extreme environments and offers opportunities for the development of space-to-ground camouflage technologies.

## Results

### Principle for space-to-ground camouflage with radiative heat dissipation

For pertinently space-to-ground camouflage, it is of crucial importance to identify the most threatening detection bands. Traveling through the whole atmosphere, the infrared signal of space objects is significantly affected by atmospheric conditions. The bands with high atmospheric transmittance and faint background sky radiation require meticulous consideration, which is pivotal for the detection capabilities of ground-based observation facilities and pose substantial threats to space objects. Concurrently, an in-depth examination of the spectral attributes of thermal radiation emitted from space objects is necessitated to ascertain the optimal radiative heat dissipation band that aligns with the demands of camouflage.

In the solar radiation band (0.4–2.5 μm), the H and K bands are the most threatening detection bands. According to the Rayleigh scattering principle, the sky performs brighter background radiation in the short-wave bands (0.4–1.5 μm) during the day (Fig. 1d), obscuring the signals from space objects. Conversely, in the long-wave bands (1.5–2.5 μm), the background sky radiation is minimal (Fig. 1d), thereby highlighting the signals of space objects. As a result, the atmospheric windows in these bands, namely the H and K bands, are optimal detection bands with the advantage of a high signal-to-noise ratio. Given the temperature range of space objects, the reflected signal of solar radiation dominates in the H and K bands, as the thermal radiation of space objects in these bands is negligible. Therefore, camouflage in the H and K bands requires to increase in the absorptivity of objects, thus reducing the reflected signal.

In the thermal radiation band (2.5–25 μm), the MWIR and LWIR bands are commonly employed for the observation of space objects. Thermal radiation emitted by space objects travels through the atmosphere, where a portion is absorbed, particularly in the non-atmospheric windows (e.g., 5–8 μm & 13–25 μm bands). The remaining radiation can be captured by ground-based observers in the MWIR and LWIR bands (Fig. 1d). In addition, the background sky radiation in the MWIR and LWIR bands is relatively weak, offering favorable observation conditions. In these two bands, the signal of space objects is predominantly thermal radiation, since solar radiation is weak enough to be neglected. Consequently, camouflage in the MWIR and LWIR bands demands to reduce the emissivity to suppress thermal radiation.

For the thermal management of space objects, the VLWIR band is strategically selected for heat dissipation. Without thermal management, space objects will experience dramatic temperature fluctuations ranging from −120 to 120 °C^63^. These extreme temperatures will damage the instruments inside the objects, which require a safe temperature range of −20 to 70 °C^52^. Given the constraints of conduction & convection being blocked and the camouflage requirements in the MWIR and LWIR bands, heat dissipation can be achieved merely through radiation within non-atmospheric windows, primarily in the 5–8 μm and 13–25 μm bands. Compared to the current radiative heat dissipation band (the 5–8 μm band), the 13–25 μm band (the VLWIR band) exhibits larger radiative power density when the temperature is below 120 °C, which is more significant within the safe temperature range (Fig. 1c). Furthermore, simulations are conducted on cubical satellites with radiative heat dissipation within the VLWIR band (I) and the 5–8 μm band (II) respectively. Radiative heat dissipation in the VLWIR band enables the satellite temperature to remain within the safe temperature range, while the temperature of the satellite that uses the 5–8 μm band for heat dissipation ultimately exceeds 70 °C (see supplement 1). Thus, the VLWIR band emerges as the leading choice for radiative heat dissipation and is requested to be endowed with a high emissivity.

To summarize, the spectral characteristics of space objects must satisfy several key criteria, as indicated by the green line in Fig. 1d: (i) high absorptivity in the H and K bands to minimize reflected signals; (ii) low emissivity within the MWIR and LWIR bands for diminishing thermal radiation signals; (iii) high emissivity in the VLWIR band to facilitate radiative heat dissipation. These design principles are critical for attaining space-to-ground infrared camouflage with radiative heat dissipation and provide a foundation for the development of advanced camouflage materials and structures.

### Structure design and measurements

**Fig. 2:**
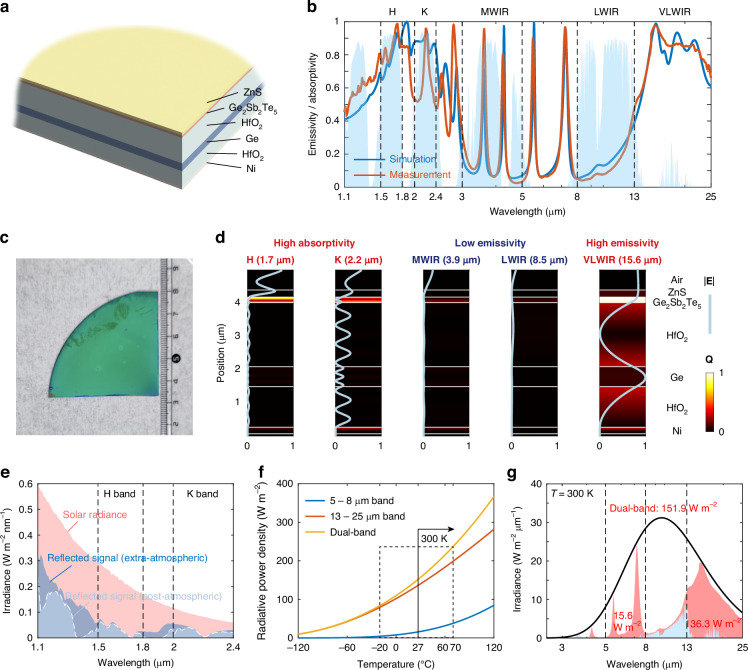
**Simulations and characterizations of the structure that enables compatibility with infrared camouflage and radiative heat dissipation**. **a** The multilayer structure utilized. **b** The simulated and measured emissivity/absorptivity spectrum of the structure. **c** The visible photograph of the fabricated structure. **d** The electric field intensity |E| and resistive loss Q distribution at various typical wavelengths in high absorptivity/emissivity bands and low emissivity bands. **e** The simulated reflected solar radiance signals within the 1.1–2.4 μm spectral range are examined both in an extra-atmospheric environment and after atmospheric transmission (post-atmospheric). **f** The simulated radiative power density through the two non-atmospheric windows (5–8 μm and 13–25 μm) as the object temperature rises from −120 °C to 120 °C. **g** The spectral distribution of thermal radiation at 300 K in outer space (red area) and after passing through the atmosphere (blue area)

Aiming at space-to-ground infrared camouflage with radiative heat dissipation, a strategy that involves the integration of subwavelength photonic films with lossy dielectric materials (see supplement 3) is adopted to elaborately tailor the spectrum. The multilayer structure, composed of ZnS (215 nm) / Ge_2_Sb_2_Te_5_ (165 nm) / HfO_2_ (1930 nm) / Ge (600 nm) / HfO_2_ (1220 nm) / Ni (120 nm) is designed to cater to the varying demands of camouflage across the H, K, MWIR, and LWIR bands (Fig. 2a), which concurrently meets the radiative heat dissipation requirement. Crystalline Ge_2_Sb_2_Te_5_ is especially selected for its intrinsic loss in the H and K bands, due to free carriers in it as a degenerate semiconductor. In addition, the top ZnS layer is employed to reduce reflection and augment absorption within the structure. The alternating structure of Ge_2_Sb_2_Te_5_/HfO_2_/Ge/HfO_2_ layers establishes highly reflective platforms in the MWIR and LWIR bands, effectively diminishing emissivity. Beyond the wavelength of 13 μm, both Ge_2_Sb_2_Te_5_ and HfO_2_ act as lossy dielectric materials, enabling high emissivity in the VLWIR band.

The absorptivity/emissivity spectrum of the multilayer structure is simulated (Fig. 2b), with the corresponding electric field intensity |E| and resistive loss Q distribution illustrated in Fig. 2d. In the H (1.7 μm) and K (2.2 μm) bands, the electric field in the structure constitutes standing waves, which meets the anti-reflection conditions of destructive interference at the interface with air, resulting in high absorption peaks. The resistance loss in the structure mainly occurs in the Ge_2_Sb_2_Te_5_ layer and the bottom Ni layer. In the MWIR (3.9 μm) and LWIR (8.5 μm) bands, the enhanced reflection conditions are established, and the electric field and resistance losses within the structure are suppressed, leading to low emissivity in these two bands. In the VLWIR band (15.6 μm), the Ge_2_Sb_2_Te_5_ and HfO_2_ are both lossy dielectric materials and intense losses occur simultaneously in the Ge_2_Sb_2_Te_5_ layer, HfO_2_ layer, and Ni layer.

The multilayer structure is fabricated on a 4-inch silicon wafer, which is segmented into 1/4 pieces for the convenience of subsequent experiments (Fig. 2c). The experimental emissivity/absorptivity spectrum of the sample, as depicted in Fig. 2b, bears a striking resemblance to the simulated spectrum. According to Eq. (1), at 300 K, the integral emissivity of the sample in the MWIR, LWIR, and VLWIR bands is 0.132, 0.142, and 0.798, respectively, which satisfies the camouflage requirements of the two detection bands and radiative heat dissipation requirement. In Eq. (1), *ε* denotes the spectral emissivity of the sample, *I*_BB_ signifies the blackbody spectral power density, and *λ1,2* represents the starting and stopping wavelengths of the integral band respectively. The numerator of the equation represents the integral radiation power of the sample in the specified band at a temperature of *T*, and the denominator represents that of the blackbody at the same temperature.

1 $$\bar{\varepsilon }={\int }_{\lambda 1}^{\lambda 2}\varepsilon \left(\lambda \right){I}_{{\rm{BB}}}\left(\lambda ,T\right)d\lambda /{\int }_{\lambda 1}^{\lambda 2}{I}_{{\rm{BB}}}\left(\lambda ,T\right)d\lambda$$

Taking the solar radiation intensity outside the atmosphere, *I*_solar_, as the reference, the integrated absorptivity (Eq. (2)) of the sample in the H and K bands can be calculated as 0.839 and 0.633, respectively. Accordingly, the irradiance spectrum of the reflected signal of the space object both outside and after passing through the atmosphere is obtained, as depicted by the dark blue (extra-atmospheric) and light blue (post-atmospheric) regions respectively in Fig. 2e. Notably, after absorbed by the sample, the intensity of the reflected infrared signal is significantly diminished relative to the solar irradiance, which vividly illustrates the effective functional camouflage performance.

2 $$\bar{\alpha }={\int }_{\lambda 1}^{\lambda 2}\alpha \left(\lambda \right){I}_{{\rm{solar}}}\left(\lambda \right)d\lambda /{\int }_{\lambda 1}^{\lambda 2}{I}_{{\rm{solar}}}\left(\lambda \right)d\lambda$$

### Radiative heat dissipation

Given the temperature range of space objects, the VLWIR band is preferred for heat dissipation, thereby mitigating thermal load and enhancing thermal camouflage. Utilizing Eq. (1), the radiative power densities of the sample in the 5−8 μm band and 13−25 μm band (the VLWIR band) at various temperatures (Fig. 2f) are derived. Within the temperature range of −120 to 120 °C, the VLWIR band accounts for the majority of the radiative heat dissipation power, while the 5–8 μm band serves as a minor supplement. At a suitable operation temperature of 300 K, which is typical for instruments on space objects, the radiative power density of the sample in the VLWIR band is 136.3 W m^−^^2^, and the combined radiative power density of both bands reaches 151.9 W m^−^^2^ (Fig. 2g). Predominating in radiative heat dissipation, the VLWIR band plays a crucial role in thermal management for space objects.

**Fig. 3:**
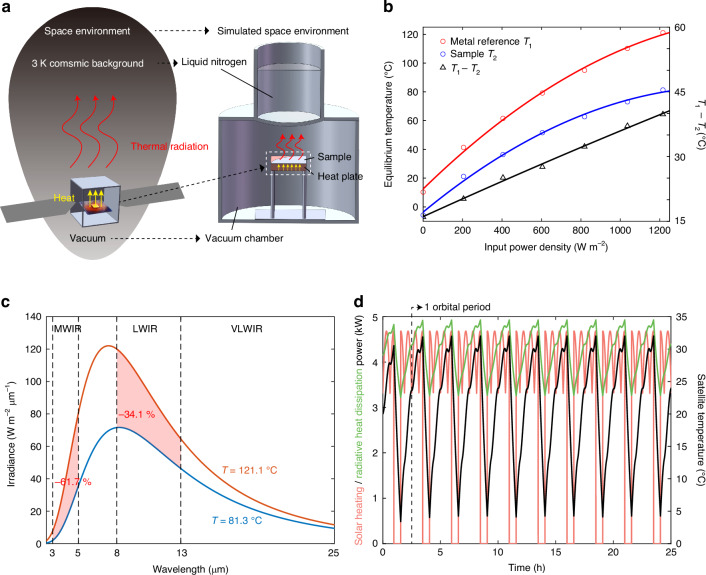
**Demonstration of radiative heat dissipation and potential space applications**. **a** The simulated space environment is constructed to demonstrate the radiative heat dissipation effect. **b** The measured equilibrium temperatures of the sample/metal reference as the input power density increases from 0 W m^−^^2^ to 1200 W m^−^^2^. **c** The reduction of thermal radiation signals in the MWIR and LWIR bands resulted from temperature decrement. **d** The variations in solar heating power (red line), radiative heat dissipation power (green line), and satellite temperature (black line) over ten orbital periods for a medium-orbit satellite. The initial temperature of the satellite is set at 20 °C and the power consumption is set at 1 kW

To investigate the heat exchange in the high-vacuum, low-temperature conditions of space, and to validate the radiative heat dissipation characteristics of the sample, a simulated space environment is constructed, utilizing the vacuum chamber in conjunction with a Dewar bottle (Fig. 3a) (supplement 4 provides comprehensive details related to the simulated space environment). The sample and a metal reference (a 120-nm-thick nickel film) of equivalent dimensions are respectively attached to a heat plate and positioned in the vacuum chamber, with support provided by nylon pillars. Above the sample, the Dewar bottle is filled with liquid nitrogen (−196 °C) and overlaid with a coating of carbon black to simulate the 3 K cosmic background. The thermal radiation emitted by both liquid nitrogen and the cosmic background constitutes less than 1% of the sample’s thermal radiation, thereby rendering the disparity in thermal radiation between them experimentally negligible. Throughout the experimental procedure, the air pressure in the chamber is stabilized at approximately 0.15 Pa. Under such controlled conditions, convective heat transfer is rendered comparatively insignificant, making the heat exchange between the sample and the surrounding environment predominantly through thermal radiation.

Under conditions of no external power input, the equilibrium temperatures of the sample and the metal reference are measured to be −5.7 °C and 10.3 °C, respectively. Notably, the sample exhibits an equilibrium temperature of 16 °C lower than that of the metal reference (Fig. 3b), demonstrating its superior radiative heat dissipation efficacy. As the input power progressively increases, a corresponding augmentation in the temperature differential between the two is discerned. When the input power density is 1200 W m^−^^2^, the metal reference attains an equilibrium temperature of 121.1 °C, whereas the sample merely reaches 81.3 °C, manifesting a substantial temperature differential of 39.8 °C (Fig. 3b). The decrement in temperature also augments thermal camouflage, evidenced by a reduction in thermal radiation signals by 61.7% in the MWIR band and 34.1% in the LWIR band, as the temperature decreases from 121.1 °C to 81.3 °C (Fig. 3c). The results prove the importance of radiative heat dissipation for space objects, enabling the expulsion of superfluous thermal energy and the preservation of thermal equilibrium in the unique environmental conditions in space. Furthermore, they corroborate the efficient radiative heat dissipation attributes of the sample.

### Space-to-ground infrared camouflage

**Fig. 4:**
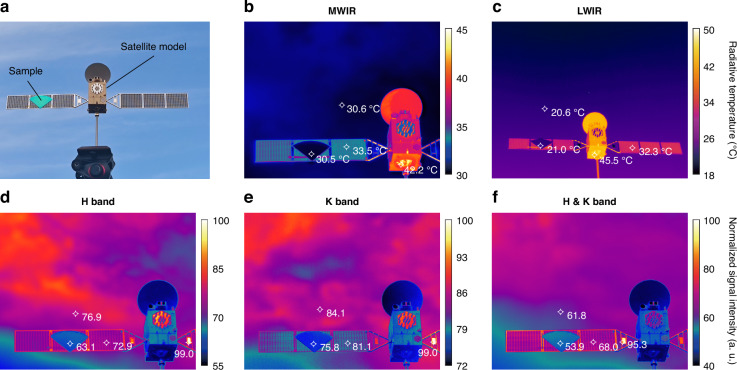
**Demonstration of space-to-ground infrared camouflage (the H, K, MWIR, and LWIR bands)**. **a** The sample is mounted onto the exterior of a satellite model, positioned outdoors in an open area. **b**, **c** The MWIR and LWIR band infrared images of the sample and the satellite model, in the form of radiative temperatures. **d**–**f** The H band, K band, and H & K band infrared images of the sample and the satellite model, in the form of normalized signal intensity

To demonstrate the infrared camouflage performance intuitively, ground-based observation scenarios are simulated, and infrared imagers are employed to capture images in various detection bands. The sample is mounted onto the exterior of a satellite model, positioned outdoors in an open area, and photographed against a celestial background (Fig. 4a) (see supplement 5 for experimental and image processing methods).

The MWIR and LWIR imagers are utilized to capture infrared images and ascertain the radiative temperatures (Fig. 4b, c). The temperature of the satellite model exceeds 40 °C, with the peak radiative temperatures recorded by the MWIR and LWIR imagers being 42.2 °C and 45.5 °C, respectively. In contrast, the sample affixed to the satellite model displays significantly lower radiative temperatures of 30.5 °C and 21.0 °C, respectively. These values closely align with those of the sky background, which are 30.6 °C and 20.6 °C, respectively, thereby illustrating its exceptional camouflage performance.

The infrared imager, when fitted with various filters, can be adaptively modified to capture infrared signals within specific bands: the H band (with a 1.5–2 μm filter), the K band (with a 2–2.4 μm filter), and concurrently in the H & K band (with a 1.5–2.5 μm filter) (Fig. 4d–f). In these designated imaging bands, while the reflected signal from the main body of the satellite model is subdued, metallic accessories affixed to it manifest a pronounced reflected signal, thereby revealing the presence of the satellite. Notably, the sample continues to demonstrate superior camouflage performance under these conditions. Across the three imaging bands, the sample exhibits a signal reduction of 36.9%, 24.2%, and 46.1%, respectively, in comparison to the metallic accessories on the satellite, thereby illustrating its ability to blend seamlessly into the sky background.

### Potential applications on space objects

Furthermore, to elucidate the potential applications on space objects, the multilayer structure is overlaid on the surface of a medium-orbit satellite in the simulation. The satellite is configured in a cubical form, with each side measuring 2 meters in length, and every surface enveloped by the aforementioned structure. The orbital altitude of the satellite is designated as 3000 km, the initial temperature is established at 20 °C, and the power consumption is set at 1 kW. The solar radiation power absorbed by the satellite exhibited periodic variations (red line in Fig. 3d), providing an additional thermal power input to the satellite.

To ascertain the efficacy of the structure in preserving the thermal equilibrium of the space object, simulations are conducted to observe the temperature fluctuations of the satellite over multiple orbital periods (simulation methods are given in supplement 1). Upon exposure to solar radiation, the cumulative heating power, incorporating both the absorbed solar energy and the thermal energy generated by the satellite itself, surpasses the heat dissipation power (green line in Fig. 3d), resulting in an elevation of the satellite’s temperature (black line in Fig. 3d). Conversely, when the satellite transitions into the Earth’s shadow, the heat dissipation power surpasses the heating power, leading to a decline in the satellite’s temperature. The fluctuations in the satellite’s temperature over ten orbital periods are computed, along with the corresponding thermal radiation power of the satellite (Fig. 3d). Notably, after 8 orbital periods, the satellite’s temperature begins to stabilize, maintaining a range between 4 °C and 32 °C. The implementation of the proposed multilayer structure effectively regulates the satellite’s temperature within the operational range of its internal instruments, demonstrating its thermal management capabilities. This approach applies to both low-orbit and high-orbit satellites as well (see supplement 6).

3 $${I}_{{\rm{obs}}}={\varOmega }_{{\rm{obs}}}{\int }_{\lambda 1}^{\lambda 2}{I}_{{\rm{spa}}}\left(\lambda \right)\tau \left(\lambda \right)d\lambda$$

The infrared signal received by the ground-based observer is also calculated to evaluate the efficacy of the infrared camouflage performance. According to Eq. (3), the intensity of the signal received by the observer, *I*_obs_, is determined by the signal intensity reflected/emitted by the satellite, *I*_spa_, the atmospheric transmittance, *τ*, and the solid angle of the aperture of the observer, *Ω*_obs_. Compared with conventional uncamouflaged satellite surfaces^53^, the surface enveloped by the multilayer camouflage structure manifests signal attenuations of 7.52 dB and 3.95 dB in the H and K bands, respectively. At the apex temperature in a stable orbital period (32 °C), the camouflaged satellite exhibits signal reductions of 6.08 dB and 7.65 dB in the MWIR and LWIR bands as well. The simulation results demonstrate the compatible functionalities of the structure in space-to-ground camouflage and thermal management for space objects, thereby unveiling its profound potential in space applications.

## Discussion

In this work, we present a novel strategy designed to achieve space-to-ground infrared camouflage with simultaneous radiative heat dissipation via a multilayer structure. Compared with previous studies on infrared camouflage, this work notably advances by coordinating the camouflage and thermal management imperatives of space objects tailored for the unique operational environment of outer space, thereby offering enhanced attributes and benefits. Firstly, camouflage in multiple ground-based infrared detection bands (the H, K, MWIR, and LWIR bands) is realized, with the effectiveness in evading infrared detection validated through simulated ground-based observation scenarios. Secondly, effective radiative heat dissipation is attained within the VLWIR band, which exhibits remarkable temperature decrements in the simulated space environment and enhancements in thermal camouflage. Thirdly, the structure is thin and robust, composed of 6 layers of inorganic material film (with an aggregate thickness of merely 4.25 μm), rendering it suitable for space applications. Finally, based on space applications, this research broadens the scope of infrared spectral manipulation to encompass the H, K, and VLWIR bands. Ultimately, this work holds significant prospects for augmenting our capabilities in space exploration and exploitation, thereby paving the way for humanity to venture into expanded realms of habitable space.

## Materials and methods

### Simulations

The absorptivity/emissivity spectrum was simulated using the transfer matrix method. Furthermore, FDTD Solutions was utilized to simulate the spatial distribution of electric field intensity. The refractive indices and extinction coefficients of the materials employed in the simulations are detailed in Supplement 2.

### Fabrication

The Ni layer was deposited onto a 4-inch silicon substrate via magnetron sputtering. Subsequently, a multilayer film composed of HfO_2_/Ge/HfO_2_ was fabricated using electron-beam (E-beam) evaporation. Following this, a Ge_2_Sb_2_Te_5_ film was deposited by magnetron sputtering, and a ZnS film was deposited using the E-beam evaporation technique.

### Spectral measurements

The infrared spectrum across the range of 1.1 to 25 μm was acquired using a Fourier-transform infrared (FTIR) spectrometer (Vertex 70, Bruker).

### Radiative intensity/temperature and absolute temperature measurements

Radiative temperature measurements were conducted using specialized instrumentation across distinct spectral bands. A Jenoptik camera was utilized to determine the radiative temperature within the LWIR band, while a Telops camera was employed for measurements in the MWIR band, as well as for signal intensity acquisition in the H, K, and combined H&K bands (using band-pass filters of 1.5–2 μm, 2–2.4 μm, and 1.5–2.5 μm, respectively). Absolute temperature measurements were obtained using thermocouples (model 5TC-TT-K-30-36, Omega).

### Air pressure

The pressure within the vacuum chamber was monitored using a Pirani vacuum gauge (VCT160, Infitech).

## Supplementary information

Supplementary information for space-to-ground infrared camouflage with radiative heat dissipation

## Data Availability

The datasets used and/or analysed during the current study are available from the corresponding author on reasonable request.
