# Supplementary material for: Space-to-ground infrared camouflage with radiative heat dissipation
Source: Light Sci Appl. 2025 Mar 26;14:137. doi: 10.1038/s41377-025-01824-y (PMC11947227; doi:10.1038/s41377-025-01824-y)
Supplement: Supplementary file 1 — Supplementary information for space-to-ground infrared camouflage with radiative heat dissipation [file 41377_2025_1824_MOESM1_ESM.docx]

Supplementary Information for

**Space-to-ground infrared camouflage with radiative heat dissipation**

*Bing Qin^1^, Huanzheng Zhu^1^, Rongxuan Zhu**^1^, Meng Zhao^1^, Min Qiu^2^, and Qiang Li^1,*^*

^1^State Key Laboratory of Extreme Photonics and Instrumentation, College of Optical Science and Engineering, Zhejiang University, Hangzhou 310027, China

^2^Key Laboratory of 3D Micro/Nano Fabrication and Characterization of Zhejiang Province, School of Engineering, Westlake University, Hangzhou 310024, China

^*^E-mail: qiangli@zju.edu.cn

**Supplement 1. Simulations of thermal management of satellites**

Simulations are conducted to observe the temperature fluctuations of the satellite with a specific spectrum over multiple orbital periods (Fig. S1). The satellite is configured in a cubical form, with each side measuring 2 meters in length. The orbital altitude of the satellite is designated as 3000 km, the initial temperature is established at 20 ℃, and the power consumption is set at 1 kW. For simulation purposes, the satellite is represented as an aluminum alloy frame structure with a mass of 2350 kg and an average specific heat capacity of 98 J kg^-1^ K^-1^.

The temperature variation of the satellite, *ΔT*, is the result of two opposite effects (equation S1): (1) heat radiation capture (primarily from Sun), *Q*_sun_ and own heat production, *Q*_sat_; (2) heat extraction by thermal radiation, *Q*_rad_.

$Q_{\mathrm{sun}}+Q_{\mathrm{sat}}-Q_{\mathrm{rad}}=cm\Delta T$ (S1)

As illustrated by equation S2, *Q*_sun_ is determined by the absorptivity of the satellite, *α*, the irradiance of the Sun, *I*_sun_, and the area irradiated by the Sun, *A*_sun_.

$Q_{\mathrm{sun}}(t)=\alpha I_{\mathrm{sun}}A_{\mathrm{sun}}(t)$ (S2)

As indicated by equation S3, *Q*_rad_ is determined by the emissivity of the satellite, *ε*, the irradiance of a blackbody, *I*_BB_*(T)*, and the surface area of the satellite, *A*_sat_.

$Q_{\mathrm{rad}}\left( t \right)=\varepsilon I_{\mathrm{BB}}\left( T\left( t \right) \right)A_{\mathrm{sat}}$ (S3)

**Fig. S1 | Schematic diagram of the simulated scenario of a cubical satellite and the temperature fluctuations of satellites with specific radiative heat dissipation bands [(I) the 13 – 25 μm band, (II) the 5 – 8 μm band].**

**Supplement 2. Refractive index and extinction coefficient of the materials**

**Fig. S2 | Refractive index (n) and extinction coefficient (k) of the materials.**

**Supplement 3. Photonic films with lossy dielectric materials**

The approach of employing photonic films incorporating lossy dielectric materials represents a strategic evolution from the conventional Ge/ZnS layered structures. By substituting select layers of Ge and ZnS with Ge_2_Sb_2_Te_5_ and HfO_2_, materials that are known for their intrinsic loss in specific spectral bands. This methodology significantly enhances emission and absorption within those bands. This innovative strategy demonstrates marked advantages over traditional Ge/ZnS layered configurations in terms of spectral manipulation efficiency.

(1) Within the H and K bands, Ge is almost lossless and the photonic film architecture primarily functions to mitigate reflections through interference effects at select wavelengths (structure S1, Fig. S3a). The absorption characteristics of the structure within the H and K bands are predominantly attributed to the underlying metallic layer. Conversely, Ge_2_Sb_2_Te_5_ exhibits high losses in the H and K bands, thereby substantially amplifying absorption efficiency in these bands. Substitution of the upper Ge layer with Ge_2_Sb_2_Te_5_ (structure S0, Fig. S3a) leads to a significant enhancement in absorptivity, from 0.514 to 0.782 in the H band and from 0.242 to 0.876 in the K band, effectively satisfying the camouflage demands for these bands. Furthermore, given Ge_2_Sb_2_Te_5_'s intrinsic loss property in the VLWIR band, its integration into the structure also yields an augmented emissivity in the VLWIR range.

(2) Within the VLWIR band, ZnS exhibits limited loss characteristics, posing challenges for ZnS-based photonic film configurations to attain high emissivity with constrained layer counts and minimal thickness (structure S2, Fig. S3b). Conversely, HfO_2_ demonstrates pronounced losses in the VLWIR band, thereby augmenting absorption in this band. Substitution of the ZnS layers with HfO_2_ layers results in a significant elevation of VLWIR emissivity from 0.4 to 0.774 (structure S0, Fig. S3b), effectively addressing the requirement for efficient radiative heat dissipation.

**Fig. S3 | Comparation of the emissivity/absorptivity spectra of photonic films with lossy dielectric materials (Ge_2_Sb_2_Te_5_, HfO_2_) and conventional materials (Ge, ZnS). (a)** The upper Ge layer is replaced with the Ge_2_Sb_2_Te_5_ layer. **(b)** Two layers of ZnS are replaced with HfO_2_.

**Supplement 4. Setup for radiative heat dissipation demonstration experiments**

Experimental demonstrations of radiative heat dissipation are conducted in the simulated space environment, achieved through the combined use of a vacuum chamber and a Dewar bottle. A 120-nm-thick nickel film served as the metal reference. The measured emissivity/absorptivity spectrum of this nickel film is presented in Fig. S4. The selection of the nickel film as a reference is motivated by two primary factors: (1) Nickel and its alloys are prevalent in aerospace applications^1,2^, making them suitable for mimicking the surface properties of space objects without covering the sample, thereby serving as an appropriate control group; (2) The nickel film exhibits extremely low emissivity within the thermal radiation bands, allowing it to act as a control group for non-radiative heat dissipation. This comparison with the radiative heat dissipation capabilities of the sample underscores the critical role of radiation in managing thermal loads for space objects.

**Fig. S4 | The measured emissivity/absorptivity spectrum of the nickel film** **reference.**

Thermal conduction, convection, and radiation in the simulated space environment are controlled to confirm the validity of the experiments.

**Thermal conduction control:** The heating apparatus is supported using hollow nylon columns, strategically chosen for their low thermal conductivity and minimal contact surface area with the heating element. This design feature substantially mitigates the effects of thermal conduction.

**Thermal convection control:** The vacuum chamber is capable of achieving a pressure reduction to 0.15 Pa, aligning with the conditions necessary for simulating the space environment. Utilizing the natural convection model specific to external conditions above a horizontally oriented plate at 20 °C^3^, calculations indicate that the convective heat transfer from the sample amounts to approximately 3.81 mW. In stark contrast, the radiative heat dissipation directed towards liquid nitrogen registers around 384 mW. Given that the convective contribution constitutes a mere 1% of the total radiative heat transfer, it is deemed negligible within the experimental context. Consequently, this level of vacuum effectively replicates the near-vacuum conditions encountered in space.

**Thermal radiation control:** To emulate the extremely low-temperature backdrop typical of the space environment, liquid nitrogen is employed. A Dewar bottle, charged with liquid nitrogen and positioned directly overhead the sample, serves this purpose. The underside of the Dewar bottle is coated with a layer of carbon black, exhibiting an emissivity near unity. Calculations reveal that the radiative heat dissipation of the sample towards the 3K space background approximates 386 mW, deviating by less than 1% from its counterpart using liquid nitrogen as the cold sink. This marginal discrepancy falls within experimental tolerance, thereby validating the use of liquid nitrogen as a practical substitute for simulating the frigid vacuum of space in thermal management studies.

A constant power source is used to supply the heat plate while a thermocouple is attached to the surface of the sample/metal reference to measure temperature. The input power is calculated by Joule’s law: *P = UI*. The size of the heat plate is 5cm×5cm, and the average power density is calculated by *P*_density_ *= P / A*.

**Supplement 5. Setup for Space-to-ground infrared camouflage experiments**

Outdoor experiments simulating space-to-ground camouflage are conducted under clear atmospheric conditions. The test sample is affixed to a satellite model, strategically positioned in an unobstructed area to circumvent infrared emissions from adjacent buildings and vegetation. Oriented towards the solar azimuth, the sample's surface facilitates the capture of sunlight reflection within the visible spectrum, enabling the identification of the solar radiation reflection path. Infrared imagers are employed to monitor infrared signals in this reflected direction. Utilizing the manufacturer's calibration curve, radiative temperatures within the MWIR and LWIR bands are ascertained, while the digital signal intensity per pixel across the H/K band is recorded by the imager's detector. To mitigate internal noise interference from the imager, control measurements are taken with the lens cap in place under identical operational parameters. Following background noise subtraction and normalization procedures, the resultant processed infrared images are derived (Fig. S5).

**Fig. S5 | Background noise subtraction and normalization** in the **(a)** H, **(b)** K, and **(c)** H&K band.

**Supplement 6. Thermal management of satellites in various orbital altitude**

The orbital altitude of a satellite significantly influences its orbital period and the duration it spends in Earth's shadow, thereby affecting the total solar radiation absorbed per orbit. Low-orbit satellites at an altitude of approximately 300 km exhibit short orbital periods (~1.5 hours), with roughly half of this time spent in Earth's shadow. Consequently, the cumulative solar energy over one orbital cycle is limited, leading to a lower maximum temperature and a smaller temperature fluctuation range (-4 to 18.6 °C) (Fig. S6a). In contrast, high-orbit satellites, positioned around 30,000 km above the Earth, experience extended orbital periods (~19 hours) and prolonged exposure times (~18 hours) to solar radiation, resulting in higher maximum temperatures. Although the proportion of time spent in shadow is minimal, the absolute duration (~1 hour) is still significant, leading to substantial radiative heat dissipation and a lower minimum temperature. As a result, high-orbit satellites encounter a broader temperature range and face greater thermal management challenges. To evaluate the thermal performance of the sample under this condition, simulations are conducted for ten orbital periods of a high-orbit satellite covered with the sample. The results indicate that under stable state conditions, the temperature varies from -8.3 to 40.6 °C within a single period (Fig. S6b), remaining within the safe operational temperature range. This demonstrates the effective thermal management capability of the sample.

**Fig. S6 | Thermal management of satellites in various orbital altitudes. (a)** The orbital altitude is 300 km. **(b)** The orbital altitude is 30000 km.

**References**

1 Smith RJ, Levis GJ, Yates DH. Development and application of nickel alloys in aerospace engineering. *Aircr Eng Aerosp Technol* 2001; **73**: 138–146.

2 Perrut M, Caron P, Thomas M, Couret A. High temperature materials for aerospace applications: Ni-based superalloys and γ-TiAl alloys. *C R Phys* 2018; **19**: 657–671.

3 Bergman TL, Lavine AS, Incropera FP, Dewitt DP. *Fundamentals of heat and mass transfer*. 8th ed. Wiley, 2017.
